# Isolation of multi‐drug‐resistant strains of *Escherichia coli* from faecal samples of dogs and cats from Harare, Zimbabwe

**DOI:** 10.1002/vms3.1472

**Published:** 2024-06-21

**Authors:** Gift Matope, Kudzai Chaima, Beauty Bande, Winnet Bare, Faith Kadzviti, Farai Jinjika, Musavenga Tivapasi

**Affiliations:** ^1^ Department of Veterinary Pathobiology, Faculty of Veterinary Science University of Zimbabwe Harare Zimbabwe; ^2^ Department of Clinical Veterinary Sciences, Faculty of Veterinary Science University of Zimbabwe Harare Zimbabwe; ^3^ Department of Veterinary Services University of Zimbabwe Harare Zimbabwe

**Keywords:** antimicrobial drugs, cats, dogs, *Escherichia coli*, multi‐drug‐resistant strains

## Abstract

**Background:**

The escalation of antimicrobial resistance (AMR) in recent years has been of major public health concern globally. *Escherichia coli* are amongst the bacteria that have been targeted for AMR surveillance due to their ability to cause infection in both animals and humans. Their propensity to produce extended spectrum beta‐lactamases further complicates the choices of treatment regimens.

**Objectives:**

To investigate the prevalence of antimicrobial‐resistance in *E. coli* strains isolated from faecal samples of dogs and cats from selected veterinary surgeries and animal shelters from Harare, Zimbabwe.

**Materials and Methods:**

A cross‐sectional study was carried out to select animals by a systematic random procedure. Faecal samples were collected for culture and isolation of *E. coli*. Their susceptibility to antimicrobial drugs was assessed using the disc diffusion method.

**Results:**

A total of 95% (133/140) of the samples from cats (*n *= 40) and dogs (*n* = 93) yielded *E. coli*. Resistance was recorded for ampicillin (45.9%), trimethoprim‐sulphamethoxazole (44.4%), nalidixic acid (29.3%), ceftazidime (15.8%) and azithromycin (12.8%), but not for gentamicin and imipenem. A total of 18% of the isolates were multi‐drug‐resistant where resistance to nalidixic acid, ampicillin and trimethoprim‐sulphamethoxazole predominated.

**Conclusion:**

We observed relatively high AMR of *E. coli* strains against ampicillin. The isolation of multi‐drug‐resistant strains of *E. coli* may signal the dissemination of resistance genes in the ecosystem of these bacteria which may have a public health impact.

## Graphical abstract

The study investigated antimicrobial resistance of *E. coli* strains isolated from dogs and cats sampled from selected surgeries and animal shelters. Antimicrobial resistance to ampicillin, trimethoprim‐sulphamethoxazole, nalidixic acid predominated, whereas no resistance was detected in gentamicin and imipenem. Multiple drug resistance to nalidixic acid, ampicillin and trimethoprim‐sulphamethoxazole being was preponderant, indicating the potential spread of resistance genes within and between species of bacteria in pet animals, humans and the environment which may augment the escalation of antimicrobial resistance.

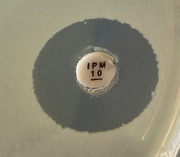

## 1 INTRODUCTION

The discovery of antimicrobial drugs in the 20th century was a major milestone in the treatment and management of infectious diseases in both humans and animals. However, the microbial agents continued to evolve and the increased use of antimicrobial drugs led to the development of antimicrobial resistance (AMR), thereby reducing their effectiveness for treatment of common bacterial infections.

The misuse of antibiotic drugs in human and veterinary medicine, combined with the extreme decline in antibiotic research, has only increased the magnitude of AMR and its impacts on global healthcare costs and outcomes (World Health Organization [WHO], 2014). The closeness of humans with pet animals and the recent globalization of the livestock industry have increased opportunities for sharing and dissemination of bacteria among animals, humans and the environment (Guinane et al., 2010). This has provided opportunities for escalating the problems of AMR. Bacteria belonging to the Enterobacterales group that includes *Escherichia coli* (*E. coli*) are ubiquitous and natural inhabitants of the gastrointestinal tracts of mammals, humans and birds but may cause severe opportunistic gastrointestinal, urinary tract and other types of extra‐intestinal infections (Harada et al., 2017). The abundance of these bacteria in animal and human populations and the environment makes them of public health interest, given their propensity to disseminate AMR genes to other closely related bacteria in the community (Bailey et al., 2010).

A notable increase of multi‐drug‐resistant phenotypes of *E. coli* and other members of the Enterobacterales, particularly to third‐generation cephalosporins, colistin and even carbapenem (used as last resort to treat serious infections) have been documented recently (Murugan et al., 2019). The reasons are not clear but related to a complex of factors such as over‐prescription and inappropriate prescription, misuse and overuse of antibiotics as growth supplements in livestock production (Aljedah, 2022). These *E. coli* phenotypes have the ability to change their clonal patterns and AMR profiles within a short period of time (Barrera et al., 2019) presumably due to great evolutionary pressure attributed to their short generation time of approximately 20 min. These bacteria have an ability to share AMR genes with other bacteria of the same or different species (Huddleston, 2014) and also appear to exhibit several mechanisms through which they develop resistance against antibiotics. The increasing problem of AMR, especially against older drugs, has led to the emergence of multi‐drug‐resistant strains of *E. coli*, posing tremendous threat to public health, animal health and the environment. This has motivated the WHO to include *E. coli* alongside other Enterobacterales amongst the list of bacteria that require new antibiotics (WHO, 2017). The escalation of multi‐drug‐resistant strains of *E. coli* transcends the species barrier because these bacteria are capable of disseminating resistance plasmids to other bacterial species. Previous reports have indicated that *E. coli* strains in a given ecosystem are closely shared between animals, humans and the environment (Mercat et al., 2016), which may facilitate the dissemination of AMR from animals to humans. Thus, the closeness of humans with pet animals provides a nexus not only of interspecies dissemination of *E. coli* strains, but also amplification of AMR. There is currently limited information of antimicrobial resistant *E. coli* phenotypes that are circulating in non‐clinical dogs and cats in (name withheld). Therefore, the objective of this study was to investigate and/or compare: (a) the prevalence and distribution of antimicrobial resistant *E. coli* phenotypes; and (b) the resistance trends on common antibiotics used in dog and cat healthcare facilities from different areas of Harare, Zimbabwe.

## 2 MATERIALS AND METHODS

### 2.1 Study areas

A cross‐sectional study was conducted at two small animal (dogs and cats) veterinary surgeries and two animal sanctuaries (animal shelters) between September 2019 and March 2020. The two surgeries serve dogs and cats (household) from affluent areas of Harare. The two animal sanctuaries provided residence to un‐owned (stray) dogs and cats that originated mainly from the less affluent areas (name of place withheld). These surgeries and animal sanctuaries were selected on the basis of willingness (verbal consent) of their management staff and the pet owners to participate in the study.

### 2.2 Sampling and sample collection

The study was conducted on randomly selected dogs (*n* = 100) and cats (*n* = 40) that were presented at veterinary surgeries either for routine examination or elective treatment or were sheltered at the animal sanctuaries. The dogs and cats were selected using systematic random sampling method collecting one faecal swab from every 5th and 10th cat and dog presented for examination, respectively. The total number of animals sampled for both species was therefore determined by the number of patients admitted to the selected premises during the study. One faecal sample was collected aseptically from each of the selected animals by the rectal swabbing method, essentially as described by Benvenuti et al. (2020). Briefly, the perineal area was aseptically washed with povidone‐iodine, followed by a wipe using a cotton swab wetted with 70% alcohol. A sterile cotton swab was then inserted into the rectum and rolled gently to collect a faecal sample. The samples were preserved in Stuart's transport medium and transported to the (name of the laboratory withheld) laboratory on cooled conditions (2–8°C) where culture and isolation was commenced within 4 h of collection. To minimize the inhibitory effect of antibiotics on bacterial cultures, only dogs and cats that were apparently healthy and were not on antibiotic treatment were eligible for sampling. Following the method adopted elsewhere, only animals that were not on antibiotic treatment for at least 6 months preceding the sampling period were included in the study (Ramos et al., 2022).

Epidemiological data such as origin of the animal (household or shelter), species (dog or cat) and sex were recorded on patient observation forms immediately after collecting the faecal samples.

### 2.3 Laboratory tests

#### 2.3.1 Culture and isolation of *Escherichia coli*

All the laboratory tests were carried out at (name of Laboratory withheld). The faecal samples were inoculated on both MacConkey agar (CM0317Oxoid) and Eosin methylene blue agar (EMB) (CM0069 Oxoid) and incubated aerobically at 37°C for 18 h. The MacConkey agar plates were examined for typical bright pink and flat lactose fermenting colonies suggestive of *E. coli*. On EMB agar, the presumptive *E. coli* colonies were large blue–black with a green metallic sheen. The definitive identification of *E. coli* isolates was based on colony morphology and lactose fermentation on MacConkey agar, Gram‐staining reaction and biochemical tests, including the presence of motility, sugar fermentation, ability to produce indole, methyl‐red and failure to produce acetoin (VP) and oxidase enzyme or utilize citrate, as described in detail by Barrow and Feltham (2023).

#### 2.3.2 Antimicrobial susceptibility testing

The agar disc diffusion method was carried out as described by the Clinical and Laboratory Standards Institute for antimicrobial susceptibility testing of bacteria (CLSI, 2023). Approximately 18‐h young cultures of pure isolates were used to inoculate culture media. The inoculum was standardized by optically matching with the 0.5 McFarland turbidity standard (corresponding to approximately 1.5 × 10^8^ cfu/mL). Suspension was then poured uniformly onto individual Mueller‐Hinton (CM0337 Oxoid) agar plates. The inoculated plates were allowed to stand for 10 min to allow excess moisture to be absorbed by the agar before placing the antimicrobial discs carefully onto the surface of the agar. The following antimicrobial discs were used: imipenem (10 µg), azithromycin (15 µg), nalidixic acid (30 µg), ceftazidime (10 µg), trimethoprim‐sulphamethoxazole (5 µg), gentamicin (120 µg) and ampicillin (25 µg). The choice of the antibiotics to use was guided by the recommendations for testing and reporting on Enterobateriales (CLSI, 2023), and also reflecting the antibiotics that are in common use in the study areas. In addition, ceftazidime, a third generation cephalosporin was included to detect ESBL‐producing *E. coli* as recommended (Kumar et al., 2014). Although increasing the panel of the tested antibiotics could have yielded additional information, the panels we selected allowed the detection of multi‐drug resistance as defined (Magiorakos et al., 2011).

Plates were inverted and incubated aerobically at 37°C for 18–24 h. The zones of inhibition were measured to the nearest a millimetre from the back of the plate. The interpretation of the size of the zones of inhibition was done with the reference to the tables produced by the CLSI (2023). The isolates were classified as susceptible, intermediately susceptible or resistant to each antimicrobial agent in the test based on the diameters of the zones of inhibition. The *E. coli* ATTC 25922 strain was used as a control during the study.

### 2.4 Data analysis

#### 2.4.1 Descriptive statistics

Data on *E. coli* isolates and antimicrobial susceptibility tests were recorded in a Microsoft Excel spread sheet where edits were done. Data analysis was carried out using Statistical Package for Social Sciences (SPSS) version 16.0 to generate descriptive statistics with respective to the prevalence of *E. coli* as well as the AMR profiles for each of the antimicrobial drugs used in the study. The chi‐square (*χ* ^2^) test was used to assess differences in prevalence and antimicrobial susceptibility of *E. coli* isolates between the species, sex and housing type, and values of *p* < 0.05 were considered significant.

## 3 RESULTS

### 3.1 Descriptive statistics

A total of 140 rectal swab samples were collected from cats (*n *= 40) and dogs (*n* = 100), and they were cultured. Of these, 95% (133/140) samples were positive for *E. coli* from 40 cats and 93 dogs, whereas 5% (7/140) had other unidentified bacteria. A total of 90% (63/70) and 100% (30/30) samples from surgeries and animal sanctuary were positive for *E. coli*, respectively, but there was no significance difference (*p *> 0.05) between the proportions.

### 3.2 Prevalence of antimicrobial resistance

The AMR profiles of the *E. coli* isolates are summarized in Table 1. All *E. coli* isolates were susceptible to imipenem (10 µg) and gentamicin (120 µg). The highest AMR in *E. coli* strains was recorded for ampicillin (25 µg) 45.9% (61/133; 95%CI: 37.6–54.1), followed by trimethoprim‐sulphamethoxazole (5 µg) 44.4% (59/133; 95%CI: 36.1–51.9). Relatively lower AMRs were observed for nalidixic acid (30 µg) 29.3% (39/133; 95%CI: 21.8–36.8), ceftazidime (10 µg) 15.8% (21/133; 95%CI: 9.8–12.8) and azithromycin (15 µg) 12.8% (17/133; 95%CI: 7.5–18.8) (Table 1). The AMR for nalidixic acid was significantly higher (*p *< 0.05) in cat than dog isolates, whereas no significant difference was observed for azithromycin, ceftazidime and trimethoprim‐sulphamethoxazole.

**TABLE 1:** Individual antimicrobial resistance (AMR) profiles *Escherichia coli* strains isolated from rectal swabs of cats and dogs (name of place withheld).

	Cat isolates (*n* = 40)		Dog isolates (*n* = 93)		^#^Total isolates (*n* = 133)	
^*^Antimicrobial drug	Number resistant	% Resistant	Number resistant	% Resistant	Number resistant	% Resistant
Ampicillin	16	40^a^ (25–55)	45	48.4^a^ (37.6–59.1)	61	45.9^3^ (37.6–54.1)
Azithromycin	5	12.5^a^ (2.5–22.5)	12	12.9^a^ (6.5–20.4)	17	12.8^2^ (7.5–18.8)
Ceftazidime	6	15^a^ (8.6–23.7)	15	16.1^a^ (8.6–23.7)	21	15.8^2^ (9.8–12.8)
Cotrimoxazole	17	42.5^a^ (27.5–57.5)	42	45.2^a^ (34.4–54.8)	59	44.4^3^ (36.1–51.9)
Gentamicin	0	–	0	–	0	–
Imipenem	0	–	0	–	0	–
Nalidixic acid	17	42.5^a^ (27.5–57.5)	22	23.7^b^ (15.1–32.3)	39	29.3^4^ (21.8–36.8)
AMR	24	60^a^ (44. 5–75.5)	72	77.4^b^ (68.8–86.0)	96	72.2 (64.5–79.9)
MDR	10	25^a^ (11.3–38.7)	14	15.1^a^ (7.7–22.4)	24	18 (11.4–24.7)

Abbreviations: AMR, antimicrobial resistance; MDR, multiple drug resistance.

* Prevalence of AMR of *Escherichia coli* isolates from dogs and cats for the same antibiotic with different superscripts are significantly different at *p *< 0.05.

^#^ Total prevalence of AMR across different antibiotics with different superscripts are significantly different at *p *< 0.05.

A total of 18% (24/133; 95%CI: 11.4–24.7) of the *E. coli* isolates were found to be resistant to three or more antimicrobial drugs (multi‐drug resistant) with two (2) isolates resistant to all five antimicrobial drugs of ampicillin, azithromycin, ceftazidime, trimethoprim‐sulphamethoxazole and nalidixic acid (data not shown). Approximately 91.7% (22/24) of these were resistant to ampicillin, whereas 79.2% (19/24) each were resistant to nalidixic acid and trimethoprim‐sulphamethoxazole (data not shown). Multiple drug resistance to nalidixic acid/ampicillin/trimethoprim‐sulphamethoxazole was most predominant with 9.8% (13/133), followed by ceftazidime/azithromycin/ampicillin 5.3% (7/133) (Table 2).

**TABLE 2:** Proportions of the total *Escherichia coli* isolates showing multiple antimicrobial resistance.

	Multi‐drug‐resistant isolates	
Antibiotics	Number resistant	% Resistant
Nalidixic acid/ampicillin/trimethoprim‐sulphamethoxazole	13	9.8
Ceftazidime/azithromycin/ampicillin	7	5.3
Azithromycin/nalidixic acid/trimethoprim‐sulphamethoxazole	2	1.5
Ceftazidime/nalidixic acid/ampicillin	1	0.7
Ceftazidime/ampicillin/trimethoprim‐sulphamethoxazole	1	0.7
Total	24	18

## 4 DISCUSSION

The observed high prevalence of faecal *E. coli* carriage in dogs and cats investigated in this study justified the choice of this organism for profiling susceptibility to antibiotics that are commonly used in the selected surgeries and the animal sanctuary. Together with other Enterobacterales, *E. coli* has been considered an ideal indicator organism for AMR and possible dissemination of resistant clones across host animals, humans and the environment, given that they are abundant in faecal matter and documented reservoirs of AMR genes in addition to being relatively easy to culture and assess for susceptibility to antimicrobial drugs compared to rare pathogenic bacteria (Joosten et al., 2020; Nyirabahizi et al., 2020; Poolea et al., 2017).

The observed high prevalence of multiple‐drug‐resistant *E. coli* strains was in agreement with previous studies in humans (Eltai et al., 2018), animals (Aasmäe et al., 2019) and the environment (Bessa et al., 2014). These enteric bacteria tend to spread easily among humans, animals, humans and the environment during which they may acquire genetic elements that confer AMR (Lynch et al., 2013). In our study, these *E. coli* strains were isolated from animals that had not undergone any antibiotic treatment for at least 6 months prior to sampling. The high prevalence of AMR to ampicillin could be attributed to the empirical use of ampicillin as a first line antibiotic in small animal veterinary practice. Resistance to beta lactam antibiotics like ampicillin is commonly developed through production of plasmid‐mediated extended spectrum beta‐lactamases (ESBLs) that help to inactivate the antibiotics (Abbas et al., 2019). Similarly, a relative higher AMR observed with trimethoprim‐sulphamethoxazole may reflect its common use in both veterinary and human medicine and concurs with previous studies in animals (Rzewuska et al., 2015; Qekwana et al., 2018) and humans (Kibret & Abera, 2011). The mode of resistance in *E. coli* to sulphonamide antibiotics primarily occurs through the mutation of structural gene encoding di‐hydro‐pteroate synthase (Sanchez‐Osuna et al., 2019) or plasmid‐mediated (Toma & Deyno, 2015).

The moderate resistance of *E. coli* isolates to nalidixic acid reported in this study has been documented in other studies (Nam et al., 2010; Vranic & Uzunovic, 2016). Nalidixic acid is a quinolones that has been used for the treatment of urinary tract infection caused by Gram‐negative bacteria but has since been replaced by newer antibiotics (Fabrega et al., 2008). Resistance to nalidixic acid has been observed to be increasing (Vranic & Uzunovic, 2016) and attributed to chromosomal mutation resulting in reduced affinity for the DNA gyrase (Fabrega et al., 2008). However, plasmid‐mediated resistance affecting the efflux pumps and DNA gyrase have also been reported (Leski et al., 2016).

In comparison to ampicillin, trimethoprim‐sulphamethoxazole and nalidixic acid, we recorded relative lower resistance to ceftazidime and azithromycin. Resistance to both azithromycin and ceftazidime were reported to be even lower (2%) byJoosten et al. (2020). Azithromycin has been used for the treatment of diarrhoeagenic *E. coli* because of its superior character, compared to other macrolides and the plasmid‐mediated resistance is low (Gomes et al., 2019). Similarly, low resistance has been reported for ceftazidime, a broad spectrum third‐generation cephalosporin used for treatment of a variety of infections (Tavio et al., 2014).

The observed lack of AMR against imipenem and gentamicin in our study is consistent with the results of other previous studies (Joosten et al., 2020). This could be because carbapenem drugs are expensive and are mainly used when they are prescribed after a culture, and sensitivity tests are performed. However, resistance to imipenem due to the production of carbapenamases (Martens & Demain, 2017) has been reported in humans (Nordmann & Poirel, 2019).

In support of other studies (Vranic & Uzunovic, 2016; Joosten et al., 2020), we recorded multiple drug resistance in *E. coli* strains predominantly against nalidixic acid, ampicillin and trimethoprim‐sulphamethoxazole. These multi‐drug‐resistant strains could potentially spread of antimicrobial resistant genes in their community of bacteria, highlighting their public health significance.

A major limitation of this study was the lack molecular‐based methods to detect the presence of resistance genes. However, the agar disk diffusion method is a well‐known procedure and is the official method used in many clinical microbiology laboratories for routine antimicrobial susceptibility testing as recognized by the CLSI. Although molecular methods provide further information on the identity of the resistance genes, the disk diffusion method offers many advantages over other methods: simplicity, low cost, the ability to test enormous numbers of microorganisms and antimicrobial agents, and the ease to interpret results provided (Balouiri et al., 2016). Although molecular based methods offer several advantages, including quick turn‐around times, greater accuracy in detecting the underlying genetic mechanism(s) for AMR (Anjum et al., 2017), they also face a challenge of implementation and harmonization, especially in low‐income countries.

## 5 CONCLUSIONS

This study revealed high AMR levels of *E. coli* from dogs and cats to ampicillin, trimethoprim‐sulphamethoxazole, nalidixic acid, varying low resistance to azithromycin and ceftazidime, but no resistance to gentamicin and imipenem. Multiple drug‐resistant strains of *E. coli* were identified, with resistance to nalidixic acid, ampicillin and trimethoprim‐sulphamethoxazole being predominant. Molecular based methods are required to investigate the types of circulating AMR genes and the role played by *E. coli* strains in disseminating these genes from pet animals to humans and the environment.

## AUTHOR CONTRIBUTIONS

Gift Matope did study design and writing of manuscript; Kudzai Chaima and Beauty Bande conducted sampling, sample testing and drafting manuscript; Winnet Bare, Faith Kadzviti and Farai Jinjika did study design and editing of manuscript; Musavenga Tivapasi did laboratory study design, supervising sample testing and editing manuscript.

## CONFLICT OF INTEREST STATEMENT

The authors declare that there are no conflicts of interest.

## FUNDING INFORMATION

None.

### ETHICS STATEMENT

The study was approved by the Faculty of Veterinary Science Higher Degrees Committee. The collection of faecal samples using the rectal swabbing method was done under the supervision of a veterinarian at both the veterinary surgeries and animal shelters. The methods of handling, restraint and after‐care treatment of animals were carried out in order to minimize stress, as recommended in the Terrestrial Animal Health Code for use of animals in research and education (OIE, 2011). The purpose of the study was explained to the animal owners and the personnel at the surgeries and animal shelters who all agreed to participate in the study.

### PEER REVIEW

The peer review history for this article is available at https://publons.com/publon/10.1002/vms3.1472.

## Data Availability

Data related to this article may be obtained from the corresponding author upon reasonable request. orcid.org/0000‐0002‐2615‐7394
